# Moderate dietary protein restriction alters the composition of gut microbiota and improves ileal barrier function in adult pig model

**DOI:** 10.1038/srep43412

**Published:** 2017-03-02

**Authors:** Peixin Fan, Ping Liu, Peixia Song, Xiyue Chen, Xi Ma

**Affiliations:** 1 State Key Laboratory of Animal Nutrition, China Agricultural University, No. 2 Yuanmingyuan West Road, Beijing 100193, China; 2 Department of Internal Medicine, Department of Biochemistry, Center for Autophagy Research, University of Texas Southwestern Medical Center, Dallas, TX 75390-9113, USA

## Abstract

This study was conducted to investigate impacts of dietary protein levels on gut bacterial community and gut barrier. The intestinal microbiota of finishing pigs, fed with 16%, 13% and 10% crude protein (CP) in diets, respectively, were investigated using Illumina MiSeq sequencing. The ileal bacterial richness tended to decrease when the dietary protein concentration reduced from 16% to 10%. The proportion of *Clostridium_sensu_stricto_1* in ileum significantly decreased, whereas *Escherichia-Shigella* increased with reduction of protein concentration. In colon, the proportion of *Clostridium_sensu_stricto_1* and *Turicibacter* increased, while the proportion of *RC9_gut_group* significantly decreased with the dietary protein reduction. Notably, the proportion of Peptostreptococcaceae was higher in both ileum and colon of 13% CP group. As for metabolites, the intestinal concentrations of SCFAs and biogenic amines decreased with the dietary protein reduction. The 10% CP dietary treatment damaged ileal mucosal morphology, and decreased the expression of biomarks of intestinal cells (Lgr5 and Bmi1), whereas the expression of tight junction proteins (occludin and claudin) in 13% CP group were higher than the other two groups. In conclusion, moderate dietary protein restriction (13% CP) could alter the bacterial community and metabolites, promote colonization of beneficial bacteria in both ileum and colon, and improve gut barrier function.

Dietary protein is the fundamental source of amino acids for livestock. However, in current animal husbandry industry, high crude protein (CP) level and imbalance of amino acids in diets result in enormous waste of protein source and severe nitrogen pollution^1^. The shortage of protein source and the nitrogen excretion environmental pollution have been becoming global serious problems. Specifically, the finishing pigs consume relatively more dietary proteins and produce more urine and feces. According to the NRC recommendation (NRC 1998), the dietary CP level is suggested at 15.5% for finishing pigs (50–80 kg) per day. However, previous studies reported that low-protein diet could decrease the concentration of ammonia-nitrogen in feces and urine of pigs^1^,^2^, but its effects on growth performance and gut health have not been well documented.

The mammalian digestive tract is colonized by a dense, dynamic and highly complex community of microorganisms composed mainly of bacteria, whose total number exceeds 10^14^ cells with thousands of individual strains^3^. Gut microbiota plays vital roles in nutrient metabolism, pathogen resistance, and development of immune system^4^,^5^. The high concentration of short-chain carbohydrates and proteins promotes the growth of bacteria in the small intestine^6^. In contrast, in the large intestine, most of the available nutrients for bacteria are derived from indigestible carbohydrates and resistant starch as well as undigested protein in diet. Then these dietary nutrients undergo microbial fermentation, resulting in the production of metabolites, such as short-chain fatty acids (SCFAs) and biogenic amines^7^. Butyrate as one of major SCFAs, is known for its physiological functions, serving as fuels for colonic epithelial cells, and inducing the proliferation of intestinal cells^8^. Both SCFAs and polyamines could improve the expression of intestinal tight junction proteins, which have contributed to enhanced integrity of intestinal barrier and a better protection against pathogen invasion^9^,^10^,^11^.

The balance of climax bacterial community has established in gut of finishing pigs, with the structure of bacterial composition remaining relatively stable. However, even after the climax community has been established, there will be dynamic changes in microbial composition that occurs in response to new colonization of microbes, inflammation stress, as well as diet^12^. The influence of different dietary CP levels on the intestinal bacterial communities have been widely studied in weaned piglets, however, the balance of intestinal microbiota has not been well established yet^13^,^14^. These studies were performed by using the traditional culture-dependent methods or low throughput culture-independent methods, and only bacteria that could be cultured *in vitro* or abundant *in vivo* were well detected. With development and application of high throughput 16 S rRNA gene sequencing, it may help to explain the underlying mechanism on comprehensive variation of intestinal bacteria caused by various concentrations of dietary protein.

In the present study, the finishing pigs were selected as the experimental model, and the effects of reducing dietary protein level by different percentage on the gut health from perspective of gut microbiota, intestinal barrier function and proliferation of intestinal stem cells were investigated. The finishing pig which is a human-sized, omnivorous animal with anatomical and physiological similarities to adult has been proposed as an alternative animal model for human^15^. Therefore, the result of our study might be a reference that suggests proper dietary protein intake for adults.

## Results

### Intestinal bacterial richness, diversity and similarity

**Figure 1:**
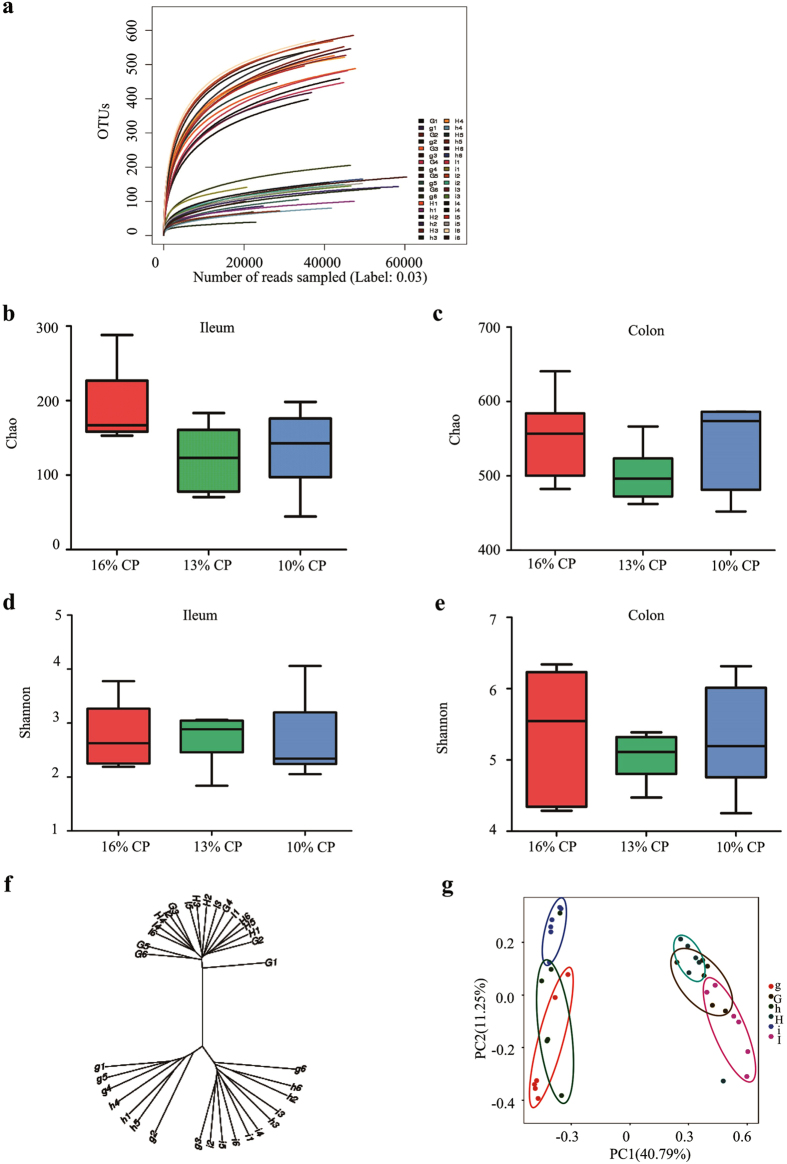
Alpha-diversity and similarity of ileal and colonic bacterial community of finishing pigs fed different concentrations of dietary protein. (**a**) Rarefaction curve for each sample (i1-i6, h1-h6, and g1-g6 are ileal digesta samples of pigs fed with 16%, 13% and 10% crude protein (CP), separately; I1-I6, H1-H6, and G1-G6 are colonic digesta samples of pigs fed with 16%, 13%, and 10% CP). (**b**) The bacterial richness in ileum estimated by the Chao 1 value. (**c**) The bacterial richness in colon estimated by the Chao 1 value. (**d**) The bacterial diversity in ileum estimated by Shannon index. (**e**) The bacterial diversity estimated by Shannon index. (**f**) Hierarchical clustering analysis of ileal and colonic bacterial community. (i1-i6, h1-h6, and g1-g6 are ileal digesta samples of pigs fed with 16%, 13% and 10% CP, separately; I1-I6, H1-H6, and G1-G6 are colonic digesta samples of pigs fed with 16%, 13%, and 10% CP). (**g**) Principal component analysis (PCA) of ileal and colonic bacterial community.

In total, after size filtering, quality control and chimera removal, 1,566,782 valid sequences were obtained, with an average of 40,141 ± 3,192 sequences per ileal sample, and 46,902 ± 1,507 per colonic sample. The overall OTU numbers classified at the distance level of 0.03 (97% similarity) were 1,015, with 398 detected in ileal samples, 920 in colonic samples, and 303 were shared by both. All samples reached a stable plateau based on rarefaction curve analysis (Fig. 1a), suggesting the sampling was sufficient for the majority of the bacterial communities.

Colonic bacterial community had much more OTU numbers, higher Chao estimate and Shannon index than ileal one (Fig. 1b,c). When the concentrations of dietary protein decreased from 16% to 13% and 10%, respectively, Chao estimate of bacterial community tended to be decreased in ileal of pigs (*P* = 0.07), while no significant difference was observed in colon. Meanwhile, Shannon index of bacterial community in either ileal or colon was barely affected by reduction of protein concentration (Fig. 1d,e). However, within-group difference of Shannon index in 13% CP group was smaller than the other two groups.

The OTU community comparisons by hierarchical clustering showed that samples from ileal and colon clustered, and most of ileal samples in 16% CP dietary treatment were separated from of those in 10% CP group, but colonic samples did not cluster clearly according to the protein concentration (Fig. 1f). The similar result could be observed in PCA plot (Fig. 1g). Ileal samples and colonic samples were apparently clustered separately. Meanwhile, both ileal and colonic samples were also clustered together based on the dietary protein concentration. The effects of decreasing protein concentration on bacterial community in both ileum and colon were also appreciable.

### Ileal bacterial community structure

**Figure 2:**
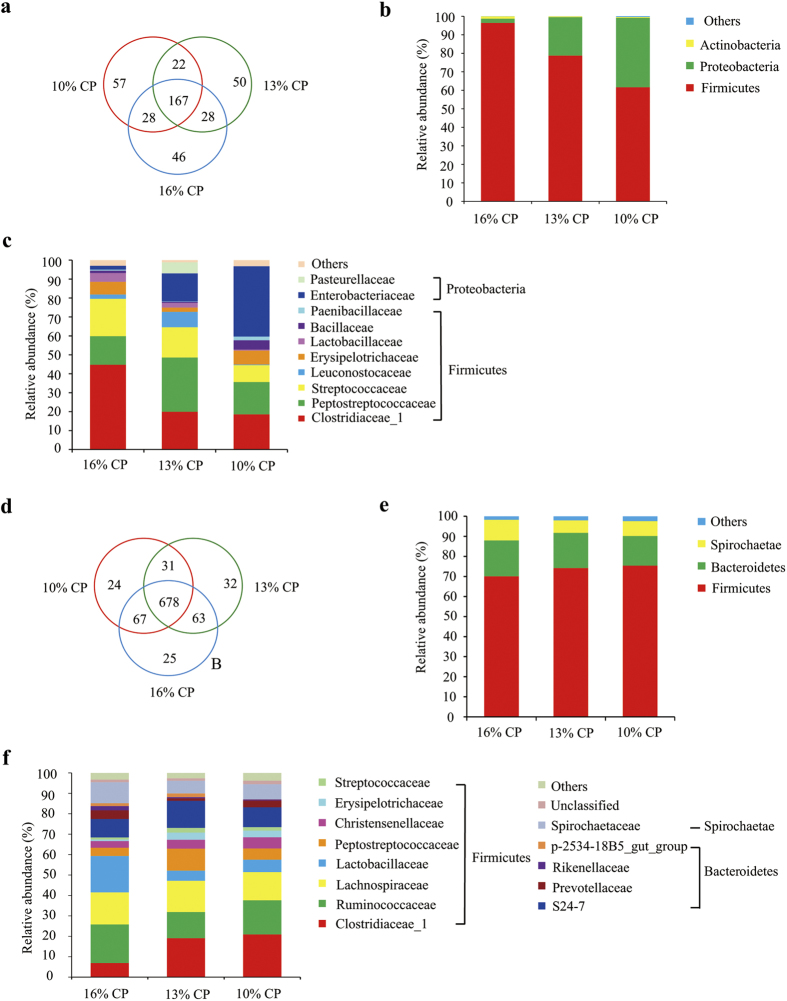
Effects of low-protein diets on ileal and colonic bacterial community structure of finishing pigs. (**a-c**): Effects of low protein diets on ileal bacterial community structure of finishing pigs. (**a**) Venn of the OTUs in different treatments. (**b**) Distribution of ileal bacteria at phylum level in finishing pigs. (**c**) Distribution of ileal bacteria at family level in finishing pigs. Phyla and families with proportion less than 1% are not listed. CP, the levels of crude protein in diet. (**d–f**): Effects of low protein diets on colonic bacterial community structure of finishing pigs. (**d**) Venn of the OTUs in different treatments. (**e**) Distribution of finishing pigs’ colonic bacteria at phylum level. (**f**) Distribution of finishing pigs’ colonic bacteria at family level. Note: Phyla and families with proportion less than 1% are not listed.

Ileal bacterial community of three groups shared about 42% OTUs shown in Venn diagram (Fig. 2a). Each group possessed similar amount of unique OTUs.

Firmicutes, Proteobacteria and Actinobacteria are dominant phyla in ileum of the finishing pigs, accounting for more than 99% of the total ileal bacterial community (Fig. 2b). However, their respective proportion varied with reduction of dietary protein concentrations. When protein concentration reduced from 16% to 13% and 10%, abundance of Firmicutes significantly decreased from 96.37% to 78.76% and 61.62%, respectively (*P* = 0.009), while abundance of Proteobacteria increased from 2.32% to 20.78% and 37.52% (*P* = 0.009). Proportion of Actinobacteria also tended to decrease when protein concentration decreased by 6 percent (*P* = 0.06). No significant differences were observed between proportions of all the 3 dominant phyla between 13% CP and 10% CP group.

At family level, Firmicutes of ileal bacteria community were mainly composed of Clostridiaceae_1, Peptostreptococcaceae, Streptococcaceae, Leuconostocaceae, Erysipelotrichaceae, Lactobacillaceae, Bacillaceae and Paenibacillaceae, while Proteobacteria consisted of Enterobacteriaceae and Pasteurellaceae (Fig. 2c). Colostridiaceae_1 was the most dominating family in 16% CP dietary group, but its proportion dropped dramatically from 47.78% to 19.94% and 18.56% after protein concentration decreased by 3 and 6 percent (*P* = 0.007). However, proportion of Enterobacteriaceae was sharply increased after protein concentration from decreased 16% to 10% (*P* = 0.008).

Down to genus levels, *Clostridium_sensu_stricto_1, Escherichia-Shigella, Streptococcus, Weissella* and *Actinobacillus* are predominant genera of Colostridiaceae_1, Enterobacteriaceae, Streptococcaceae, Leuconostocaceae, and Pasteurellaceae, respectively (Table 1). Their proportional variation with the reduction of protein concentration mainly led to the change of proportion at family level. In addition, about 15–30% ileal bacteria community were unidentified at genus level.

### Colonic bacterial community structure

About 74% OTUs were shared among 3 groups of colonic bacterial community (Fig. 2d). And each group owned similar amount of unique OTUs.

Firmicutes, Bacteroidetes, and Spirochaetae were 3 major bacterial phyla in colonic contents of finishing pigs, accounting for more than 97% of the total colonic bacterial community (Fig. 2e). Their respective proportion did not significantly alter with reduction of dietary protein concentration.

At family level, Firmicutes in colon were mainly composed of Clostridiaceae_1, Ruminococcaceae, Lachnospiraceae, Lactobacillaceae, Christensenellaceae, Erysipelotrichaceae and Streptococcaceae (Fig. 2f). Proportion of Clostridiaceae_1 (*P* = 0.03) and Erysipelotrichaceae (*P* = 0.004) in 16% CP group were both significantly lower than those in 13% and 10% CP group. And 13% CP group had a larger proportion of Peptostreptococcaceae than other 2 groups (*P* = 0.01). The proportion of other bacterial families were not significantly influence by protein concentration. Bacteroidetes mainly consisted of S24-7, Prevotellaceae, Rikenellaceae and p-2534-18B5_gut_group. Only the proportion of Rikenellaceae was significantly affected by protein concentration in diets, decreasing after protein concentration reduced from 16% to 13% and 10% (*P* = 0.048). Spirochaetaceae is the primary family of Spirochaetae, and no significant difference of its proportion was observed among 3 groups.

Down to genus level, *Clostridium_sensu_stricto_1, Turicibacter* and *RC9_gut_group* are dominant genera of Clostridiaceae_1, Erysipelotrichaceae and Rikenellaceae, separately (Table 2). Their proportion varied consistently with their affiliated family. However, approximate half of colonic bacterial community were unidentified at genus level.

### Concentration of intestinal SCFAs

The concentration of acetate and valerate in ileal content decreased significantly when dietary protein concentration decreased from 16% to 13% and 10% (*P* < 0.01, Table 3), but concentration of propionate, butyrate and isovalerate in ileal did not differ among 3 groups, and isobutyrate was not detected in the ileal content. As for colonic content, when dietary protein concentration decreased from 16% to 13%, concentration of acetate, propionate and isobutyrate declined significantly (*P* < 0.01), and when dietary protein concentration continued to decrease at 10%, concentration of butyrate and isovalerate also dropped down (*P* < 0.01). But no significant difference in concentration of colonic valerate was observed among 3 groups.

### Concentration of intestinal biogenic amines

When dietary protein concentration reduced by 3 percent, concentration of all the 5 biogenic amines in ileal content, and putrescine, histamine, and spermidine in colonic content were significantly decreased (*P* < 0.05, Table 4), but when the concentration of dietary protein reduced to 10%, only concentration of ileal histamine and colonic methylamine continued to decrease significantly (*P* < 0.05).

### Intestinal morphology

**Figure 3:**
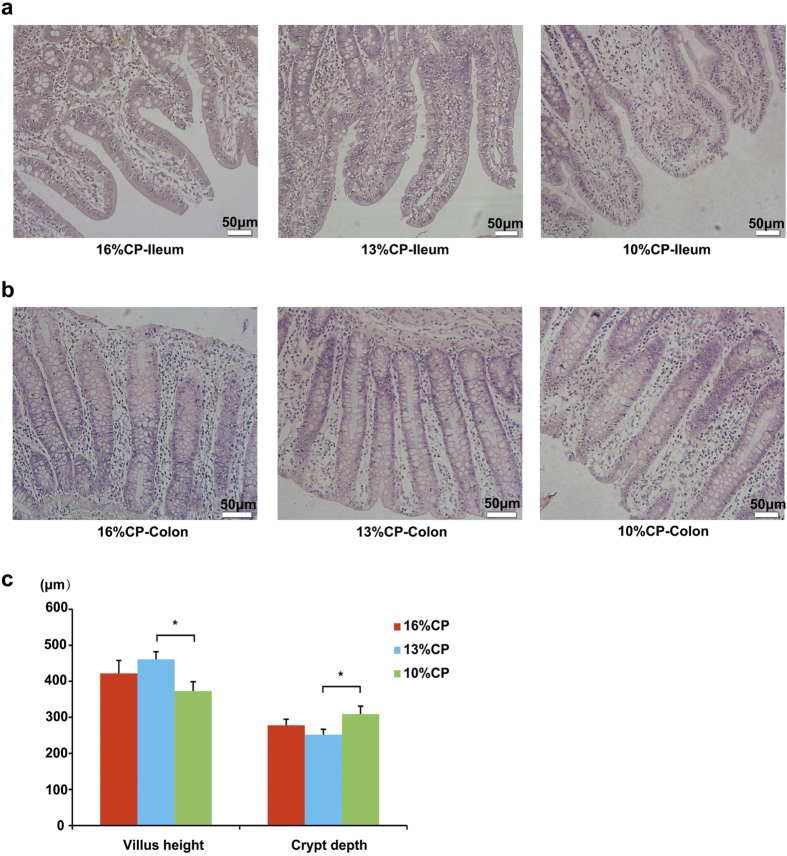
Effects of low protein diets on the intestinal mucosal morphology of finshing pigs (the stained sections were photographed at 100x magnification). CP, the levels of crude protein in diet. (**a**) Ileal morphology observation of different dietary groups. (**b**) Colonic morphology observation of different dietary groups. (**c**) Villus height and crypt depth were measured. Values are means±standard deviations (n = 6). Star indicates a significant difference (*P* < 0.05).

Effects of low-protein diets on the intestinal morphology of finishing pigs were shown in Fig. 3. Ileal morphology of finishing pigs in 16% CP group and 13% CP group were more integrate than those in 10% CP group (Fig. 3a), but no apparent damage was observed in colon among different groups (Fig. 3b). Compared to 16% and 13% CP dietary treatments, ileal tissue in 10% CP dietary treatment showed shorter villus height (*P* = 0.04) and deeper crypt depth (*P* = 0.03, Fig. 3c).

### Expression of intestinal tight junction proteins and biomarkers of intestinal stem cells

**Figure 4:**
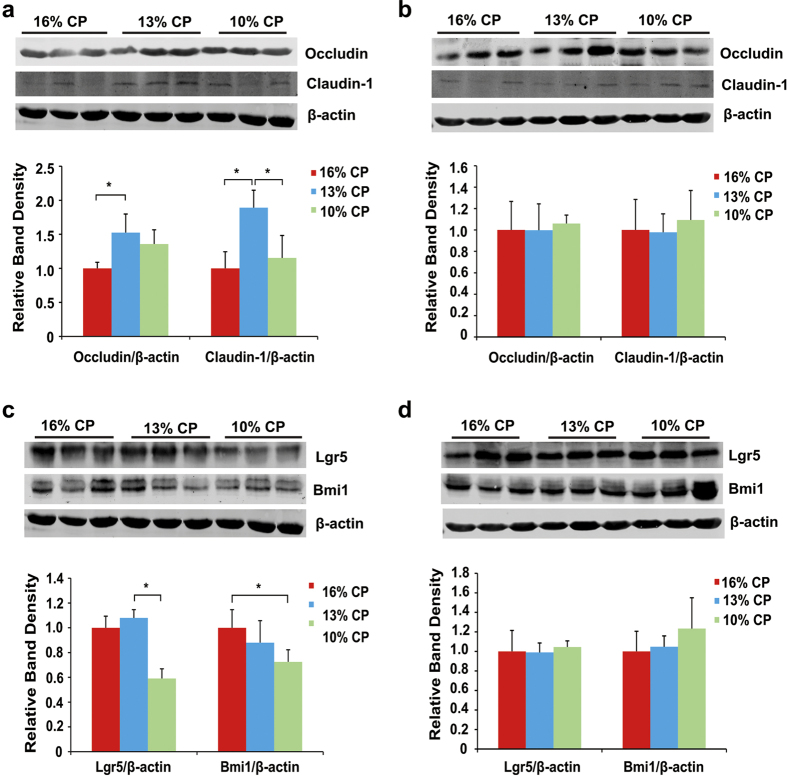
Effects of low protein diets on expression of intestinal tight junction proteins and biomarkers of intestinal stem cells of finishing pigs. CP, the levels of crude protein in diet. (**a-b**): Expression of occludin and claudin-1 in ileal tissue (**a**) and colonic tissue (**b**). Values are means ± standard deviations (n = 3). Stars indicate a significant difference (*P* < 0.05). (**c-d**): Expression of Lgr5 and Bmi1 in ileal tissue (**c**) and colonic tissue (**d**). Values are means ± standard deviations (n = 3). Star indicates a significant difference (*P* < 0.05).

Effects of low-protein diets on expression of intestinal tight junction proteins and biomarkers of intestinal stem cells were shown in Fig. 4. Expression of ileal claudin-1 in 13% CP group was significantly higher than 16% CP and 10% CP group (*P* < 0.01), and reducing the dietary protein concentration by 3 percent from 16% increased the expression of occludin (*P* = 0.04). However, decreasing dietary protein concentration had no significant influence on expression of colonic tight junction proteins. Leucine-rich repeat-containing G-protein-coupled receptor 5 (Lgr5) and bmi1 polycomb ring finger oncogene (Bmi1) are biomarkers of intestinal stem cells. No significant difference was observed in expressions of ileal Lgr5 and Bmi1 between 16% CP and 13% CP group, but expression of Lgr5 in 10% CP group was significantly lower than the other two groups (*P* = 0.02), and Bmi1 in 10% CP group was lower than 16% CP group (*P* = 0.04). But there was no significant difference in biomarkers of colonic stem cells among 3 groups.

## Discussion

The low-protein diets in animal husbandry should alleviate the serious problems of global nitrogen excretion environmental pollution and the shortage of protein sources, but its influence on the gut micro-environment and intestinal health are still unknown. In the present study, low-protein diets altered the intestinal bacterial community of finishing pigs. Chao estimate indicated the bacterial richness and Shannon index reflected the bacterial diversity. When dietary CP concentration drop by 3 to 6 percent, ileal bacterial richness tended to decrease, suggesting that the protein deficiency may inhibit the growth of intestinal bacteria. Though bacterial diversity was slightly affected, the samples of three groups were clustered separately. And a separation between ileal samples of 16% and 10% CP groups could be best observed from PC1 and PC2. Therefore, reduction on the level of dietary protein concentration was indicated to affect the gut microbiota, especially to ileum, which was consistent to the changes of ileal bacteria community among 3 groups.

Results of studies with amino acid-metabolizing bacteria in small intestine indicate that bacteria belonging to the *Clostridium, Streptococcus* and *Escherichia-Shigella* play an important role in amino acid utilization in animals^16^,^17^,^18^,^19^. In the present study, it indicated that *Clostridium_sensu_stricto_1* was the absolutely the predominant genus in the ileum of finishing pigs fed high protein diet, but its proportion sharply decreased when the dietary CP level reduced, which was mainly due to the shortage of protein substrate for fermentation, and this was consistent with a previous study^20^. Likely, proportion of *Streptococcus* tended to decrease in the same way. However, abundance of proteolytic bacteria *Escherichia-Shigella* dramatically increased in the low-protein dietary groups. Members of *Escherichia-Shigella* prefer to live in the weakly alkaline environment, thus greater amount of SCFAs generated from high-protein diet may partly inhibit the proliferation the *Escherichia-Shigella*^15^. Members of *Clostridium* and *Escherichia-Shigella* were reported to be associated with necrotizing enterocolitis more frequently than other groups^21^, and 16% CP group and 10% CP group had greater total proportion of these bacteria than the 13% CP group, indicating finishing pigs fed with high-protein diet or extremely low-protein diet which had much higher proportion of *Clostridium* and *Escherichia-Shigella* in gut content may be more susceptible to infection of necrotizing enterocolitis. In addition, proportion of *Actinobacillus, Weissella* and Peptostreptococcaceae tended to increase when dietary protein concentration decreased from 16% to 13%. *Actinobacillus* could cause respiratory disease in pigs, and its proportion also increased in fecal samples of primary biliary cirrhosis patients^22^. However, early intervention with sodium butyrate had a benefit role in the health of neonatal piglets with down regulated ileum inflammatory cytokine and increased proportion of *Actinobacillus*^23^, indicating some members of this genus may inhibit the intestinal inflammation. *Weissella* could also exert both beneficial and harmful effects depending on the specific species. And as reported, Peptostreptococcaceae is usually considered as normal commensal bacteria, and its proportion is higher in gut microbiota of health animals than those patients with dysbiosis of the intestinal microbiota^24^, indicating Peptostreptococcaceae helps maintain the gut homeostasis.

In consistent with healthier pattern of ileal bacterial community in 13% CP group, ileal expression of two important intestinal tight junction proteins occludin and claudin-1 were both up regulated when the dietary protein concentration decreased by 3 percent. The intestinal epithelial barrier maintained segregation between luminal microbial communities and the mucosal immune system^25^. This barrier is primarily regulated by a well-organized system referred to as the tight junction, which is comprised of several unique proteins including the trans-membrane protein occludin, junction adhesion molecule, and members of the claudin-1 family as well as linker proteins such as zonula occludens protein-1^26^. In our study, the increased expression of ileal tight junction may be regulated by varied bacterial community, but further research is needed to verify the promotion.

Reduction of dietary protein concentration from 16% to 10% dramatically damaged mucosa of ileal morphology with lower ratio of intestinal villus height to crypt depth that impact on absorptive capacity for nutrients. The adverse change in ileal morphology is likely related to high proportion of *Escherichia-Shigella* and protein deficiency to maintain the gut architecture of intestinal epithelium. Meanwhile, expression of biomarkers of intestinal stem cell (ISC) were also lower in 10% CP group. The intestinal epithelial barrier is initiated by the intestinal stem cell (ISC) niche that gives rise to the differentiated cell types, including Paneth cells, enteroendocrine cells, goblet cells, and absorptive enterocytes or colonocytes^27^. Lgr5+ ISC at the base of crypt are wedged between Paneth cells, and “+4 stem cells” reside right above the Paneth cells, which are two predominant ISCs. Lgr5 and Bmi1 are the markers of the two types of ISC, respectively^28^,^29^. In the present study, the expression of Lgr5 and Bmi1 were decreased significantly when the dietary protein concentration reduced from 16% to 10%, indicating proliferation of ileal stem cells requires adequate protein content. A previous study reported that ISC proliferation was induced by the commensal *Lactobacillus* due to its Nox1-mediated induction of ROS in the intestinal epithelium of mice^30^,^31^, which is accordant with our study that the decreased expression of biomarkers of ISC from 16% to 10% CP group was accompanied by decline of the *Lactobacillus* proportion. Whether other varied bacterial communities, such as *Clostridium_sensu_stricto_1* and *Escherichia-Shigella* can influence proliferation of ISC demands to be further investigated.

Compared to the ileum, colonic bacterial community did not dramatically vary with the reduction of dietary protein concentration, especially at phylum level. But down to family and genus level, there were still some apparent differences among three groups. The proportion of *Clostridium_sensu_stricto_1* in colon increased significantly with the reduction of dietary protein concentrations, which was opposite in ileum. Since bacteria composition varied in ileum and colon, *Clostridium_sensu_stricto_1* in colon of 16% CP group may be inhibited by other bacteria. The proportion of *RC9_gut_group* belong to Rikenellaceae decreased when dietary protein concentration declined, suggesting adequate nitrogen source is indispensable for proliferation of *RC9_gut_group*. Meanwhile, proportion of Turicibacter, which is positively correlated to colitis^32^, increased with the reduction of dietary protein concentrations. Notably, the colonic proportion of Peptostreptococcaceae in 13% group was higher than the other two groups, which was in consistent with what we found in ileum of pigs. This alteration may be due to the preference of moderate protein concentration of this bacteria family, but more likely, profiting from competition of other bacteria. However, in contrast to ileum, no significant damage on colonic morphology, expression of tight junction proteins and biomarkers of ISCs were observed in low-protein diet groups, which may be partly due to the mild change of colonic bacteria community.

SCFAs, mainly acetate, propionate and butyrate, are produced by fermentation of undigested dietary proteins and fibers. Isobutyrate and isovalerate, also known as branched-chain fatty acids (BCFAs), are coming from deamination of branched-chain amino acids, valine and leucine by bacteria, thus are considered markers of protein fermentation^19^. The present data demonstrated that concentrations of SCFAs in both ileum and colon were decreased by low-protein diets, suggesting a weaker bacterial fermentation caused by nitrogen reduction. However, the drop of SCFAs concentration with the reduction of dietary protein in ileum was not as dramatic as that in colon, especially for propionate, butyrate and BCFAs. This is mainly due to the low abundance of bacteria in ileum, so the reduced protein concentration may still meet the requirement of bacteria that produce these SCFAs. But in colon, all the SCFAs except for valerate decreased when dietary protein concentration decreased from 16% to 10%, but concentration of butyrate and BCFAs in 13% CP group did not significantly differ from those in 16% CP group. SCFAs have profound effects on metabolism and gut health. Acetate and propionate are energy substrates for peripheral tissues, and butyrate is preferentially used as an energy source by colonic epithelial cells^33^. Therefore, the low-protein diets may decrease the energy produced by bacteria fermentation for host. However, recent study found that acetate could promote obesity via a gut–brain–β-cell axis^34^, indicating that high concentration of dietary protein diet may raise the risk of obesity by increasing the level of acetate in both ileum and colon. Besides, interactions of acetate and propionate with GPR43^35^, as well as activation of GPR109A by butyrate are proposed to have an important role in inducing their anti-inflammatory effects via the modulation of T_Reg_ cells^36^. Though no apparent damage of colon was discovered in low-protein diet groups, more immune parameters are needed to detect to investigate the effects of protein reduction on gut health more comprehensively.

In addition, low-protein diets decreased concentration of biogenic amines in ileum and colon of pigs in the present study, indicating intensity of protein fermentation in intestine was decreased. Only concentration of colonic cadaverine, the metabolite of lysine was not reduced significantly, and this may be due to the supplementation of lysine in the low-protein diets. Biogenic amines are low molecular organic polycations produced by decarboxylation of amino acids by intestinal microbiota such as *Bacteroides, Clostridium, Enterobacterium* and *Streptococcus*, and play important roles in cell physiology by regulating gene expression, signal transduction, ion channel function, DNA and protein synthesis, and apoptosis through binding with RNA, DNA, nucleotide triphosphate, proteins, and other negatively charged molecules^34^. However, high concentrations of biogenic amines can induce adverse reactions such as nausea, headaches, rashes and changes in blood pressure^38^.

The effects of dietary protein restriction on growth performance also been recorded. The weight gain of finishing pigs in 13% CP group (mean of final body weight: 97.88 kg) showed no significant (*P* > 0.05) difference with those in 16% CP group (mean of final body weight: 101.43 kg), whereas the weight gain 10% CP group (mean of final body weight: 94.02 kg) were significantly (*P* < 0.05) lower than those in 16% CP group. These data indicate that moderate dietary protein restriction (13% CP) may meet the requirement of finishing pig growth in commercial pig farms. The moderate dietary protein restriction (13% CP) may be preferred by pork industry, which introduces a new strategy to improve the health of animal gut health, because it did not damage the intestinal morphology, and could improve the epithelial barrier function and develop a healthier pattern of ileal bacterial community.

## Conclusions

In conclusion, moderate reduction of dietary protein concentration (13% CP) could improve the bacterial community structure in both ileum and colon of finishing pigs, especially increase the proportion of Peptostreptococcaceae, and enhance the ileal barrier function. In contrast, over-reduction of dietary protein (10% CP) would damage gut bacteria community and ileal morphology, as well as inhibit intestinal stem cell proliferation. Intestinal concentrations of SCFAs and biogenic amines decreased with reduction of dietary protein concentration.

## Materials and Methods

### Ethics

All management and experimental procedures were in accordance with the animal care protocols approved by the China Agricultural University Animal Care and Use Ethics Committee (CAU20151010-2). All experimental protocols were approved, and the methods were carried out in accordance with the relevant guidelines and regulations.

### Animals, diets and sample collection

A total of 18 finishing pigs (Duroc × Landrace × Yorkshire, as examples for adult pigs, initial body weight 62.30 ± 1.00 kg) selected from 4 litters were used in the study. For purposes of this research, this sample size was considered adequate to give conclusive results for tests of hypotheses associated with the objectives of this study. These piglets were delivered vaginally and fed continuously in our experimental farm. The pigs were randomly assigned to 3 treatments with 6 pigs per treatment on the basis of similar body weight. Pig fed diets included normal dietary protein concentration group (16%), low dietary protein concentration group (13%), and extremely low dietary protein concentration group (10%), respectively. The dietary treatment met the National Research Council (NRC, 2012) nutrient specifications for 51 to 100 kg BW pigs. Diets were formulated to contain similar digestible energy (DE) content and equal standardized ileal digestible contents of essential amino acids lysine, methionine plus cysteine, threonine and tryptophan. The composition and nutrient content of diets is shown in Supplementary Table 1.

All pigs were housed with one pig per pen and *ad libitum* throughout the whole experiment. All pigs were slaughtered on 50 days later. Ileal and colonic contents were collected and then stored at −80 °C for genomic DNA isolation, analysis of SCFAs and biogenic amines. Ileal and colonic segments were collected for observation of intestinal morphology and detection of protein expression.

### DNA extraction and PCR amplification

DNA extraction of intestinal contents was performed according to the instructions of a DNA Stool Mini Kit (Qiagen, Hilden, Germany). The bacterial universal V3-V4 region of the 16 S RNA gene was amplified according to PCR bar-coded primers 338 F (5′-ACTCCTACGGGAGGCAGCA-3′) and 806 R (5′-GGACTACHVGGGTWTCTAAT-3′). PCR was performed in a total of 20 μL volume, containing 1 × FastPfu Buffer, 250 μM dNTP, 0.1 μM each of the primer, 1 U FastPfu Polymerase (Beijing TransGen Biotech, Beijing, China) and 10 ng template DNA. PCR was performed at 95 °C for 2 min, followed by 30 cycles of 95 °C for 30 s, annealing at 55 °C for 30 s, 72 °C for 30 s and a final extension at 72 °C for 5 min.

### Illumina MiSeq sequencing

PCR products were detected using 2% agarose gels electrophoresis, purified with AxyPrep DNA Purification kit (Axygen Biosciences, Union City, USA). The PCR products were visualized on agarose gels and were quantitatively determined using QuantiFluor-ST Fluoremeter (Promega, Wisconsin, USA) and PicoGreen dsDNA Quantitation Reagent (Invitrogen, Carlsbad, USA). Purified amplicons were pooled in equimolar and paired-end sequenced (2 × 300) on an Illumina MiSeq platform (Allwegene, Beijing, China) according to the standard protocols. And the raw data were uploaded to NCBI SRA Database, with the SRA accession: SRP090426.

### Bacterial data processing

The sequencing data were subjected to bioinformatics analysis. Raw FASTQ files were de-multiplexed and quality-filtered using QIIME (version 1.17) with the following criteria: (i) The 300-bp reads were truncated at any site that obtained an average quality score of <20 over a 10-bp sliding window, and the truncated reads shorter than 50 bp were discarded; (ii) exact barcode matching, two nucleotide mismatch in primer matching, and reads containing ambiguous characters were removed; (iii) only overlapping sequences longer than 10 bp were assembled according to their overlapped sequence. Reads that could not be assembled were discarded. Operational taxonomic units (OTUs) with 97% similarity cutoff were clustered using UPARSE (version 7.1), and chimeric sequences were identified and removed using UCHIME. The rarefaction analysis based on Mothur v.1.21.1 was conducted to reveal the diversity indices, including the Chao, Shannon, and coverage indices. The hierarchical clustering analysis was performed using the Primer 6 software (Primer-E Ltd., UK). Principal component analysis (PCA) was performed with Canoco 4.5. Venn diagrams were implemented by Venn Diagram, and community figures were generated using R tools according to the data from document “tax.phylum.xls, tax.family.xls, tax. genus.xls”.

### Detection of SCFAs in ileal and colonic contents by gas chromatography

The concentrations of SCFAs were determined by a gas chromatographic method according to the procedures of Wang *et al*. (2015) with modifications^36^. Briefly, about 1.5 g of thawed ileal and colonic digesta was suspended in 1.5 mL of distilled water in a screw-capped tube. The entire sample was centrifuged at 15,000 × g for 10 min at 4 °C. Then 1 mL of supernatant was collected to an ampoule and mixed with 200 μL of metaphosphoric acid. The ampoules were placed in an ice bath for 30 min and centrifuged again for 10 min. The sample was injected into a HP 6890 Series Gas Chromatograph (Hewlett Packard, PA, California) equipped with a HP 19091N-213 column with 30.0 m × 0.32 mm i.d. (Agilent, Santa Clara, USA). The injector and detector temperatures were 185 °C and 210 °C, respectively. Each sample was analyzed in 3 replications.

### Determination of amines in ileal and colonic digesta by HPLC

The concentrations of methylamine, spermidine, cadaverine, putrescine and histamine in digesta were determined by HPLC with a method adapted from Fan *et al*. (2016) with modifications^37^,^38^. Briefly, 0.2 g ileal and colonic digesta was weighed into a 2-mL a screw-capped tube, 1 mL trichloroacetic acid solution was then added, and the mixture was homogenized for 10 min. After that, the mixture was centrifuged at 3600 × g for 10 min at 4 °C. The pooled supernatant was mixed with an equal volume of n-hexane for 5 min, and the organic phase was discarded and the water phase was re-extracted using the same procedure. A portion of 20 mL internal standard was added to the pretreated sample, together with 1.5 mL saturated sodium bicarbonate solution, 1 mL dansyl chloride, and 1 mL NaOH, and heated at 60 °C for 45 min with occasional gentle inverting. Then the 100 μL ammonia was added into the mixture to stop the reaction. The solution was kept in the 40 °C water bath and the acetone was vaporized under nitrogen blowing. Finally, the sample was extracted twice with 3 mL diethyl ether. The combined extracts were dried under nitrogen flow and the residue was re-dissolved in acetonitrile for injection. HPLC analysis was performed using an ammonium acetate-acetonitrile gradient elution program, on an Agilent 1200 series system equipped with a dual low-pressure gradient pump, an auto sampler and a column compartment, as well as variable wavelength detector (VWD). And the column was a reversed-phase ZORBAX 80 A Extend-C18 (4.6 mm × 250 mm; 5 μm) (Agilent, Santa Clara, USA). The two solvent reservoirs contained acetonitrile and 0.2 mol/L ammonium acetate. The flow rate was 1.0 ml/min and the temperature was set at 30 °C. The wavelength was set at 260 nm for VWD. Each sample was analyzed in 3 replications.

### Intestinal morphology

Samples of ileal and colonic tissues from each finishing pig were immediately fixed in polyformaldehyde for intestinal morphological determination. The tissues were dehydrated and embedded following standard procedures^39^. The tissues in paraffin block were cut into 4-μm sections and stained with haematoxylin and eosin, specimens were examined by using a regular BX-51 microscope (Olympus). Representative photographs of the intestinal morphology of the ileum and colon were collected using Visitron Systems (Puchheim). The villus height and crypt depth were measured and analysed using NIS-Elements BR Software, Version 2.20 (Nikon).

### Extraction of protein and immunoblotting

Total protein was extracted from the intestinal tissues using lysis buffer (150 mM NaCl, 1% Triton X-100, 0.5% sodium deoxycholate, 0.1% SDS, 50 mM Tris-HCl at pH 7.4, plus a protease inhibitor cocktail purchased from Applygene, Beijing, China). Briefly, 0.02 g of each frozen intestinal segment was powdered under liquid nitrogen, and lysed in lysis buffer containing protease inhibitors. The lysed samples were centrifuged at 10,000 × g for 5 min at 4 °C and the supernatant was collected. Total protein concentrations were determined using a BCA Protein Assay Kit (Pierce, Rockford, USA). Equal amounts of proteins (40 μg) were electrophoresed on SDS polyacrylamide gel, and proteins were electrophoretically transferred onto nitrocellulose membrane (Millipore, Bedford, USA). These were then blocked in Tris-buffered saline containing 0.05% Tween-20 (TBS-T) and 5% non-fat milk (1 h at room temperature), and incubated (overnight at 4 °C) with primary antibodies against claudin-1, Lgr5 (Santa Cruz Biotechnology, Santa Cruz, USA), occludin, Bmi1 (Abcam, Cambridge, United Kingdom), and β-actin (Cell Signaling Technology, Danvers, USA). Membranes were then washed with TBS-T 3 times and then incubated with the appropriate IRDye^TM^ 800-conjugated secondary antibodies in the dark (1 h at room temperature). Following another 3 washes with TBS-T, signals were detected using the LI-COR Infrared Imaging System and quantified with Odyssey software.

### Statistical analysis

The sample size of six is appropriate, and met the requirement of the statistical power analysis, based on our previous studies and others using animals^40^,^41^. In addition, Anosim (analysis of similarity) and Amova (analysis of molecular variance) were conducted to justify the statistical power of 6 pigs per group, using the microbiota data. The results showed that there are significantly different within each group (*P* < 0.01), both in ileal and colonic digesta, which indicate that the number of each group is appropriate. Data analysis were performed using the statistical software SAS Version 9.2., and were subjected to simple one-way ANOVA with Bonferroni multiple range test. Differences at *P* < 0.05 were considered significant.

## Additional Information

**How to cite this article:** Fan, P. *et al*. Moderate dietary protein restriction alters the composition of gut microbiota and improves ileal barrier function in adult pig model. *Sci. Rep.* **7**, 43412; doi: 10.1038/srep43412 (2017).

**Publisher's note:** Springer Nature remains neutral with regard to jurisdictional claims in published maps and institutional affiliations.

## Supplementary Material

Supplementary Information

## Figures and Tables

**Table 1:** Effects of low protein diets on proportion of ileal bacteria of finishing pigs at genus level (%)^1,2^.

Classification levels of bacteria			Diet			SEM	*P value*
Phylum	Family	Genus	16% CP	13% CP	10% CP		
Firmicutes	Clostridiaceae_1	*Clostridium_sensu_stricto_1*	44.76^a^	19.92^b^	18.41^b^	4.21	<0.01
	Streptococcaceae	*Streptococcus*	19.54	15.93	8.29	2.99	0.10
	Leuconostocaceae	*Weissella*	2.29	6.04	0.32	1.81	0.06
	Erysipelotrichaceae	*Turicibacter*	6.60	2.23	7.35	1.19	0.17
	Lactobacillaceae	*Lactobacillus*	4.51	2.44	0.53	1.00	0.28
	Bacillaceae	*Bacillus*	1.32	0.60	4.92	1.2	0.30
	Paenibacillaceae	*Paenibacillus*	0.47	0.22	1.87	0.46	0.30
Proteobacteria	Enterobacteriaceae	*Escherichia-Shigella*	1.06^c^	10.9^b^	35.39^a^	4.89	<0.01
		*Klebsiella*	0.84	3.29	1.15	0.90	0.50
	Pasteurellaceae	*Actinobacillus*	0.00	5.87	0.03	1.16	0.08
—	—	*unidentified*	15.51	31.37	17.91	3.07	0.14

Note:^1^Genera with proportion less than 1% are not listed.

Values are means, n = 6. SEM: standard error of the mean.

^a,b^Different superscript within a row means significantly different (*P* < 0.05).

**Table 2:** Effects of low protein diets on proportion of colonic bacteria of finishing pigs at genus level (%)^1,2^.

Classification levels of bacteria			Diet			SEM	*P* value
Phylum	Family	Genus	16% CP	13% CP	10% CP		
Firmicutes	Clostridiaceae_1	*Clostridium_sensu_stricto_1*	6.86^b^	19.00^a^	20.78^a^	2.40	<0.01
	Lactobacillaceae	*Lactobacillus*	17.84	4.98	6.12	6.02	0.16
	Lachnospiraceae	*Blautia*	3.21	4.15	5.26	0.52	0.28
		*Roseburia*	0.48	1.08	1.22	0.28	0.54
	Erysipelotrichaceae	*Turicibacter*	0.52^b^	3.31^a^	3.06^a^	0.42	<0.01
	Streptococcaceae	*Streptococcus*	1.03	2.28	1.65	0.34	0.35
	Ruminococcaceae	*Ruminococcus*	2.97	0.94	1.02	0.59	0.29
		*Oscillibacter*	1.89	1.18	1.83	0.22	0.36
		*Flavonifractor*	1.47	0.88	1.35	0.13	0.13
Bacteroidetes	Prevotellaceae	*Prevotella*	2.18	0.38	1.64	0.54	0.40
	Rikenellaceae	*RC9_gut_group*	1.82	0.55	0.46	0.16	0.05
Spirochaetae	Spirochaetaceae	*Treponema*	10.30	6.23	7.40	1.91	0.70
—	—	*unidentified*	42.67	50.66	42.02	1.97	0.14

Note: ^1^Genera with proportion less than 1% are not listed.

Values are means, n = 6. SEM: standard error of the mean.

^a,b^Different superscript within a row means significantly different (*P* < 0.05).

**Table 3:** Effects of low protein diets on SCFAs concentration in intestinal contents of finishing pigs^1^.

Item	Diets			SEM	*P* value
	16% CP	13% CP	10% CP		
Ileal content					
Acetic acid (mg/g)	0.97^a^	0.63^b^	0.37^c^	0.070	<0.01
Propionic acid (mg/g)	0.53	0.56	0.54	0.045	0.50
Isobutyric acid (mg/g)					
Butyric acid (mg/g)	0.13	0.11	0.11	0.006	0.09
Isovaleric acid (mg/g)	0.02	0.02	0.02	0.001	0.27
Valeric acid (mg/g)	0.007^a^	0.005^b^	0.002^c^	0.001	<0.01
Colonic content					
Acetic acid (mg/g)	1.54^a^	1.28^b^	0.82^c^	0.081	<0.01
Propionic acid (mg/g)	0.78^a^	0.59^b^	0.37^c^	0.044	<0.01
Isobutyric acid (mg/g)	0.07^a^	0.05^b^	0.03^c^	0.005	<0.01
Butyric acid (mg/g)	0.38^a^	0.33^a^	0.24^b^	0.018	<0.01
Isovaleric acid (mg/g)	0.16^a^	0.15^a^	0.12^b^	0.005	<0.01
Valeric acid (mg/g)	0.09	0.10	0.08	0.003	0.16

Note: ^1^Values are means, n = 6. SEM: standard error of the mean.

^a,b,c^Different superscript within a row means significantly different (*P* < 0.05).

**Table 4:** Effects of low protein diets on biogenic amines concentration in intestinal contents of finishing pigs ^1^.

Item	Diets			SEM ^1^	*P* value
	16% CP	13% CP	10% CP		
Ileal content					
Methylamine (μg/g)	16.96^a^	10.09^b^	9.67^b^	0.944	<0.01
Cadaverine (μg/g)	60.62^a^	40.30^b^	41.85^b^	2.676	<0.01
Putrescine (μg/g)	50.61^a^	33.78^b^	36.70^b^	2.037	<0.01
Histamine (μg/g)	85.95^a^	68.87^b^	54.98^c^	3.452	<0.01
Spermidine (μg/g)	61.61^a^	39.85^b^	29.42^b^	4.136	<0.01
Colonic content					
Methylamine (μg/g)	17.58^a^	13.88^ab^	11.17^b^	0.773	<0.01
Cadaverine (μg/g)	30.28	27.43	26.84	2.045	0.79
Putrescine (μg/g)	45.48^a^	21.81^b^	22.57^b^	2.796	<0.01
Histamine (μg/g)	49.59^a^	39.02^b^	41.91^b^	1.720	0.02
Spermidine (μg/g)	36.84^a^	24.23^b^	20.47^b^	2.089	<0.01

Note: ^1^Values are means, n = 6. SEM: standard error of the mean.

^a,b,c^Different superscript within a row means significantly different (*P* < 0.05).
