# Supplementary material for: Moderate dietary protein restriction alters the composition of gut microbiota and improves ileal barrier function in adult pig model
Source: Sci Rep. 2017 Mar 2;7:43412. doi: 10.1038/srep43412 (PMC5333114; doi:10.1038/srep43412)
Supplement: Supplementary Information [file srep43412-s1.docx]

**Moderate dietary protein restriction improves the composition of gut microbiota and ileal barrier function in adult pig model**

**Peixin Fan ^1^, Ping Liu ^1^, Peixia Song ^1^, Xiyue Chen ^1^, and Xi Ma ^1,2,^***

^1^ State Key Laboratory of Animal Nutrition, China Agricultural University, No. 2 Yuanmingyuan West Road, Beijing 100193, China.

^2^ Department of Internal Medicine, Department of Biochemistry, Center for Autophagy Research, University of Texas Southwestern Medical Center, Dallas, TX 75390-9113, USA

*** For correspondence. E-mail: maxi@cau.edu.cn.**

**Supplementary Table 1. The composition and nutrient content of diets（%, as-fed basis）**

| Items | Dietary CP level | | |
| --- | --- | --- | --- |
|  | 16% | 13% | 10% |
| Ingredients^1^ |  |  |  |
| Corn | 67.00 | 78.36 | 87.40 |
| Soybean meal | 23.76 | 15.00 | 5.50 |
| Wheat bran | 6.00 | 3.00 | 2.00 |
| Soybean oil | 0.88 | 0.90 | 1.71 |
| L-Lysine HCl, 98.5% | 0.01 | 0.27 | 0.55 |
| DL-Methionine, 98% | 0.00 | 0.00 | 0.09 |
| L-Threonine, 98% | 0.00 | 0.06 | 0.19 |
| L-Tryptophan, 98% | 0.00 | 0.01 | 0.06 |
| Dicalcium phosphate | 0.50 | 0.55 | 0.65 |
| Limestone | 0.55 | 0.55 | 0.55 |
| Salt | 0.30 | 0.30 | 0.30 |
| Premix^2^ | 1.00 | 1.00 | 1.00 |
| Total | 100.00 | 100.00 | 100.00 |
| Nutrient levels^3^ |  |  |  |
| Digestible energy (MJ/kg) | 14.20 | 14.20 | 14.20 |
| Crude protein | 16.30 | 13.17 | 10.26 |
| SID^4^ Lysine | 0.72 | 0.72 | 0.73 |
| SID^4^ Methionine + Cystine | 0.50 | 0.42 | 0.43 |
| SID^4^ Threonine | 0.56 | 0.50 | 0.49 |
| SID^4^ Tryptophan | 0.17 | 0.13 | 0.13 |
| SID^4^ Arginine | 0.94 | 0.70 | 0.44 |
| SID^4^ Histidine | 0.39 | 0.31 | 0.22 |
| SID^4^ Isoleucine | 0.60 | 0.45 | 0.30 |
| SID^4^ Leucine | 1.32 | 1.13 | 0.91 |
| SID^4^ Phenylalanine | 0.71 | 0.57 | 0.41 |
| SID^4^ Valine | 0.61 | 0.50 | 0.36 |
| Calcium | 0.52 | 0.50 | 0.51 |
| Total phosphorus | 0.45 | 0.40 | 0.38 |
| Starch | 43.71 | 49.97 | 55.22 |
| Neutral Detergent Fiber | 11.33 | 10.18 | 9.37 |
| Acid Detergent Fiber | 4.34 | 3.69 | 3.14 |

Note: ^1^L-Lysine, DL-Methionine, L-Threonine, L-Tryptophan were provided by Health & Nutrition of Evonik Industries AG, Germany.

^2^Provied per kilogram of complete diet: vitamin A, 3,800 IU; vitamin D_3_, 800 IU; vitamin E, 9 mg; vitamin K_3_, 1 mg; vitamin B_2_, 2 mg; vitamin B_6_, 1.2mg; vitamin B_12_, 10μg; niacin, 10mg; biotin, 50 μg; folate,0.4mg; Mn, 3 mg; Zn, 80 mg; Fe, 80 mg; Cu, 5 mg; Se, 0.25 mg; I, 0.14 mg.

^3^All nutrient levels except digestible energy were analyzed.

^4^SID: Standard ileal digestible.
